# The psychological significance of female traditional initiation rites among the Bakopa community of South Africa: a qualitative approach to the understanding of *koma ya basadi*

**DOI:** 10.3389/fsoc.2025.1458428

**Published:** 2026-01-14

**Authors:** Mmakotsedi Magampa, Thelmah Maluleke, Rudzani Marry Mhlari, Anastasia Julia Ngobe, Lehlogonolo Makola, Abongile Pindo, Alinah Segobye, Tholene Sodi

**Affiliations:** 1 Impact Centre, Human Sciences Research Council, Pretoria, South Africa; 2 Research and Development Division, University of Limpopo, Mankweng, South Africa; 3 Division of Psychology, University of Mpumalanga, Nelspruit, South Africa; 4 Department of Psychology, University of South Africa, Pretoria, South Africa; 5 Public Health, Societies and Belonging (PHSB), Human Sciences Research Council, Cape Town, South Africa; 6 Botswana Human Resource Development Council, Gaborone West, Botswana; 7 Mental Health and Society, University of Limpopo, Mankweng, South Africa

**Keywords:** female traditional initiation, psychological significance, Bakopa women, qualitative study, cultural identity, South Africa

## Abstract

**Introduction:**

Female traditional initiation rites are deeply entrenched in the cultural fabric of many societies, representing profound rites of passage marking the transition from girlhood to womanhood. These ceremonies have important psychological, cultural, and social significance for the initiates and their communities. Referred to as “*koma ya basadi*” among Ba-Kopa, these rites encompass a series of rituals, teachings, and ceremonies designed to prepare young girls for the roles and responsibilities they will assume as adult women within their communities. Despite the documented significance of these rites, they remain misunderstood, and their psychological significance remains under-researched. This study aimed to explore the psychological importance of traditional female initiations among the Bakopa women of the Sekhukhune district of Limpopo Province.

**Methods:**

Purposive sampling was used to select 16 women from the Ba-Kopa community who had undergone *koma ya basadi* for in-depth individual interviews. These interviews were complemented by two group discussions: one focus group with six participants and one dyadic discussion with two participants. The group discussions were conducted in two separate villages approximately 20 km apart, to capture localized perspectives within the community. All data were analyzed using ATLAS.ti software, which facilitated systematic coding and thematic analysis.

**Results:**

The findings revealed that the initiation rites play a crucial role in maturing initiates, contributing to their identity formation and instilling qualities such as perseverance and tolerance.

**Discussion:**

The study underlines the importance of preserving and respecting cultural practices like traditional female initiation. It highlights the need for further research to understand their implications for individual and community wellbeing. These findings have significant implications for policymakers, healthcare providers, and community leaders in promoting cultural sensitivity and supporting the holistic development of young women.

## 1 Introduction

Initiation rites have long served as vital conduits for transmitting cultural values, beliefs, and behavioural norms across generations in many African societies (Lebese et al., 2022). Among various ethnic groups, these rites not only mark the transition from childhood to adulthood but also function as structured systems for socialization, identity formation, and the reinforcement of communal values (Nanegbe, 2016). While the initiation of boys has been widely documented, female initiation particularly outside the context of surgical interventions, has often received limited scholarly attention or has been misinterpreted through biomedical or moralistic lenses. In response to this gap, this study explores the psychological and social significance of *koma ya basadi* [female traditional initiation] practiced among the Bakopa people in Limpopo Province, South Africa.

Anthropological frameworks have long recognized the structural and symbolic dimensions of initiation. Van Gennep (1960) conceptualized rites of passage as a three-stage process separation, liminality, and reintegration that marks an individual’s transition across social boundaries. Turner (1967) expanded this model by examining the liminal phase as a period of ambiguity, symbolic death, and transformation, during which social hierarchies are suspended, and communal bonds (communitas) are strengthened. Later, Barth (1987) emphasized the dynamic nature of initiation rituals, arguing that such practices are historically contingent and responsive to changing socio-political and economic contexts. While early anthropological analyses focused predominantly on male initiation, more recent scholarship has begun to apply these frameworks to women’s initiation, viewing such rites as critical spaces for transmitting gendered knowledge, moral instruction, and cultural identity (Johnson, 2011; Kangwa, 2011; Shoko, 2009).

Among the Bakopa, *koma ya basadi* [female traditional initiation] is a highly symbolic and pedagogical process traditionally conducted in three phases. The first, *Go rupa*, begins with a preview ritual (leroleng) and prepares girls, referred to as *byale*, for seclusion and instruction. Historically lasting 2 months (now often shortened to 2 weeks), this stage involves physical endurance activities, cultural education, and symbolic rituals intended to instill discipline, obedience, and emotional resilience. The second phase, *Botsho* (meaning “black” or mourning), represents the liminal stage in which initiates undergo a symbolic death of childhood. During this period, they adhere to strict moral codes while living in seclusion under the supervision of elder women. Instruction emphasizes gender roles, community responsibilities, and the emotional demands of adulthood. The final stage, *Boramaswaile*, marks reintegration. Introduced through the *motjielo* ritual which symbolizes the initiate’s rebirth into womanhood. Girls are adorned in ceremonial attire and honored in public celebrations that affirm their new social status. They are metaphorically likened to donkeys, symbols of obedience and hard work and to men, denoting strength, resilience, and the capacity for leadership within the household.

Although Bakopa initiation rites are non-surgical and distinct from practices such as female genital alteration, they remain surrounded by secrecy and are often misunderstood. Dominant discourses especially those rooted in biomedical and human rights perspectives frequently portray female initiation as disempowering or harmful. For example, Tamale (2008) critiques such rites in Uganda as mechanisms of gendered control, while Schroeder et al. (2022) emphasize their potential to reinforce patriarchal norms around sexuality and domesticity. However, such critiques may oversimplify the lived experiences of women who view these rites as empowering and transformative. Among the Bakopa, *koma ya basadi* [female traditional initiation] is widely regarded by participants as a rite of passage that offers psychological preparation, a sense of belonging, and a moral framework for navigating adulthood. In this study, psychological significance refers to participants’ own interpretations of emotional growth, identity formation, resilience, and social preparedness acquired through the rites.

This study therefore contributes to a growing but still limited body of work on women’s initiation rites by reframing them not as biomedical anomalies or instruments of social repression, but as meaningful cultural institutions. These rites facilitate gendered socialization, intergenerational knowledge transmission, and the reinforcement of communal values (Barth, 1987; Kangwa, 2011; Shoko, 2009). Moreover, they serve as culturally embedded forms of psychosocial support, fostering identity development, emotional resilience, and social connectedness (Nyeseh Ofori and Mohangi, 2024; Sewu, 2023). While much of the literature on African initiation has focused on male rites or controversial practices, this study centers women’s own narratives of initiation to offer a more nuanced understanding of their transformative potential.

## 2 Methodology

### 2.1 Sampling and data collection processes

Prior to conducting interviews and group discussions, the researchers obtained ethical clearance from the Turfloop Research Ethics Committee at the University of Limpopo (Ethical clearance certificate number: TREC/282/2017: P.G.). Additionally, formal permission was sought from the relevant traditional authorities, namely Kgoši Matsepe and Kgoši Rammupudu, in the areas where the participants were located. Before conducting interviews, participants were briefed on the study’s objectives and assured that their involvement was voluntary. The researchers ensured that participants fully comprehended the study’s purpose and role in it, as well as the recruitment process. This facilitated participants’ understanding of the study and enabled them to make informed decisions regarding their participation. Informed consent forms were provided to participants for review and signature before their involvement.

A qualitative research design was employed to purposively recruit 16 women who had undergone traditional female initiation, ensuring that all participants had direct experience with the initiation process (Creswell and Creswell, 2017; Frechette et al., 2020). Inclusion criteria required participants to be women aged 16–90 from the Bakopa community, either by birth or marriage, who had participated in traditional female initiation regardless of the duration or depth of their involvement. Exclusion criteria included women younger than 16 or older than 90, women who had not undergone traditional initiation, and all men.

In addition to the individual interviews, two focus group discussions (including one dyadic discussion) were conducted to capture broader community perspectives. This combination of methods allowed for the exploration of both personal narratives and shared experiences, offering a comprehensive understanding of the significance and variation in traditional female initiation practices (Guest et al., 2013; Thompson Burdine et al., 2021).

### 2.2 Key informants interviews and focus groups discussion

We initiated the study with focus group and dyadic discussions to uncover shared meanings, generational perspectives, and variations in initiation experiences through dynamic, group-based reflection. These discussions provided a space for participants to compare experiences, reflect collectively, and enrich the data with cultural and emotional nuance (Morgan, 1996). During these sessions, participants were invited with open-ended prompts such as:

*“How do you think initiation practices have changed over time?”*

*“What do you believe is the purpose of these rites today?”*

These prompts encouraged broad-ranging discussions that informed the thematic direction of the subsequent interviews.

Following the group discussions, we conducted structured interviews with 16 women who had undergone traditional female initiation. Structured interviews are a well-established qualitative technique that ensures systematic data collection through predetermined questions, enabling consistency across participants and robust thematic comparison (Adeoye-Olatunde and Olenik, 2021). The interviews sought to elicit detailed personal narratives and explore participants’ beliefs, values, and reflections on initiation. The interview and focus group discussion (FGD) guides were developed through a combination of literature review, cultural context, and informal consultations with community members. Key themes were informed by existing scholarship on initiation practices, gendered rites of passage, and indigenous knowledge systems. In addition, preliminary discussions were held with two local women who had undergone initiation and were familiar with customary practices; their feedback helped ensure the relevance and cultural sensitivity of the questions. The guides were then piloted with two participants from a nearby, but distinct, community to assess clarity, flow, and appropriateness. Minor adjustments were made based on this pilot, particularly in rephrasing culturally specific terms and ensuring open-ended questions allowed participants to speak freely about their experiences.

Each interview followed a standardized format covering key themes, guided by prompts such as:

*“Can you describe your experience during the initiation?”*

*“What lessons or values were emphasized to you?”*

This combination of methods allowed us to capture both collective insights from group dialogue and individual depth from personal narratives.

All participants were women from the same cultural background who had undergone initiation. However, there were differences in roles and perspectives: focus group participants were generally older and often served as community leaders or mentors guiding younger women, while the interview group included both recent initiates and older participants. Though united by a common cultural tradition, their experiences differed in aspects such as timing, content, and mentorship.

### 2.3 Data analysis

A total of 18 transcripts were analyzed: 16 individual interviews, one focus group discussion, and one dyadic discussion. All audio-recorded data were transcribed verbatim in the original language, which was *Sekopa* [which is a dialect of Sepedi] spoken within the Bakopa community.

Following transcription, the data were backtranslated into English to ensure accessibility and comprehension for all members of the research team, particularly those not fluent in Sekopa. This process helped preserve the fidelity of participants’ meanings while making the data suitable for collaborative analysis. Special care was taken to retain the cultural and contextual integrity of key expressions and idioms during translation.

All qualitative data were analyzed using ATLAS.ti software, which facilitated a systematic and rigorous thematic analysis process. A coding framework was developed to identify and organize emerging themes and sub-themes. Two researchers independently coded an initial subset of transcripts to identify preliminary patterns. They then met to compare codes, resolve discrepancies, and collaboratively refine the coding scheme. This consensus coding approach was applied across the full dataset to ensure consistency and analytical rigor.

Thematic development was an iterative process, guided by continual engagement with the data. As more transcripts were coded, themes were refined, expanded, or reorganized to better capture the complexity and depth of participants’ experiences. The use of ATLAS.ti enabled efficient management of the data and linking of codes to relevant excerpts, supporting a traceable and transparent analytical process. This approach allowed the researchers to uncover nuanced insights and relationships, contributing to a comprehensive understanding of the psychological and social dimensions of *koma ya basadi*.

Data saturation was monitored as an ongoing process throughout data collection. Saturation was considered reached when no new themes, categories, or insights emerged from successive interviews or focus group discussions, and when participant responses began to show redundancy. This point of informational redundancy was used as a guiding principle to conclude data collection (Guest et al., 2006). Field notes and iterative coding were reviewed after each session to assess the depth and variation of emerging data. Once subsequent interviews no longer provided novel information relevant to the research questions, saturation was deemed to have been achieved.

#### 2.3.1 Researcher positionality

The research team recognizes the importance of positionality in shaping all stages of qualitative research, including data collection, interpretation, and representation.

The lead researcher is a black African woman and an insider, a member of the Bakopa community with personal familiarity with the cultural practices under study. This positionality enabled rapport and trust-building with participants, while also requiring ongoing reflexivity to avoid projecting assumed shared meanings. The second researcher is an academic with no direct ties to the community but brings extensive experience in qualitative research and gender studies in African contexts. This outsider perspective contributed critical distance and helped to balance cultural insight with analytical objectivity.

Together, these positionalities enhanced the study’s credibility, reflexivity, and cultural sensitivity, strengthening both the rigor and the authenticity of the analysis.

## 3 Results

### 3.1 Participants demographic information

As shown Table 1, participants aged 63–90 experienced initiation rituals spanning from 1945 to 1970, when they were aged between 15 and 18 at the commencement of their initiation process. None of the participants had formal education and were pensioners at the time of the study. Within this cohort, seven individuals were widowed and their initiation lasted for 6 months, whereas one individual who was married underwent initiation for 3 months.

**Table 1:** Characteristics of study participants: focus group discussions (FGD).

Participant	Age	Marital status	Initiation year/regiment	Duration (in months)	Age @initiation	Education level	Occupation
P1 (FGD)	86	Widowed	1949	6	17	None	Pensioner
P2 (FGD)	90	Widowed	1945	6	16	None	Pensioner
P3 (FGD)	81	Widowed	1954	6	18	None	Pensioner
P4 (FGD)	70	Widowed	1965	6	16	None	Pensioner
P5 (FGD)	72	Widowed	1965	6	18	None	Pensioner
P6 (FGD)	63	Married	1970	3	15	None	Housewife
P1 (DD)	81	Widowed	1954	6	18	None	Pensioner
P2 (DD)	85	Widowed	1949	6	16	None	Pensioner

As shown in Table 2, most participants were pensioners (*n* = 10) aged between 71 and 83 who participated in initiation ceremonies from 1954 to 1965. They were predominantly widowed, underwent a six-month initiation process, lacked formal education, and were initiated aged between 11 and 19. Following this group were four unemployed or student participants, aged between 23 and 33, who completed secondary education, were unmarried, and initiated between the ages of 14 and 19, with a duration of 1 month. Subsequently, there was a participant with a degree employed as a social worker, aged 44, married, and initiated at 13, with a duration of 2 months. Lastly, there was a 23-year-old participant who completed secondary education and worked as a personal assistant, married and initiated at the age of 9, with a duration of 1 month.

**Table 2:** Characteristics of study participants: key informants interviews (KII).

Part-no	Age	Marital status	Year of initiation	Duration (in months)	Age @initiation	Education level	Occupation
1	23	Unmarried	2009	1	14	Metric	Unemployed
2	23	Married	2004	1	9	Metric	PA
3	24	Unmarried	2004	2	10	Metric	Student
4	30	Unmarried	2001	1	12	Metric	Unemployed
5	33	Unmarried	1996	1	11	Metric	Unemployed
6	45	Married	1985	2	13	Degree	Soc Worker
7	71	Married	1965	6	18	None	Pensioner
8	72	Widowed	1965	6	19	None	Pensioner
9	74	Widowed	1960	6	16	None	Pensioner
10	75	Widowed	1960	6	17	None	Pensioner
11	75	Widowed	1954	6	11	None	Pensioner
12	76	Widowed	1960	6	18	None	Pensioner
13	78	Widowed	1954	6	14	None	Pensioner
14	78	Widowed	1954	6	14	None	Pensioner
15	82	Widowed	1954	6	19	None	Pensioner
16	83	Widowed	1954	6	18	None	Pensioner

### 3.2 Emerging themes

Following the data analysis process, three themes emerged as follows: (i) *Koma* as a maturation process; (ii) *Koma* as a catalyst for the formation of cultural identity and social exclusion for the uninitiated; and finally, (iii) *koma* inculcates perseverance and tolerance.

#### 3.2.1 *Koma* as a maturation process/maturity

Maturity is a multifaceted trait that encompasses emotional, intellectual, and behavioural development, leading to responsible and balanced decision-making. It involves navigating complex situations with wisdom, composure, and integrity, reflecting a deeper understanding of oneself and others. Mature individuals demonstrate resilience in the face of adversity, showing grace under pressure and a willingness to learn from their experiences. Furthermore, maturity involves taking ownership of one’s actions, accepting accountability for mistakes, and continuously striving for self-improvement. It is a hallmark of personal growth and development, reflecting a journey toward self-awareness.

The excerpts from the participants shed light on the perceived impact of female traditional initiation, commonly known as *“koma,”* on the mental and psychological development of initiates. Participants unanimously assert that the initiation process instills a sense of maturity and mental balance that distinguishes initiated individuals from the uninitiated. This sentiment is echoed throughout the discussion, with participants emphasizing the transformative nature of the initiation experience. They describe how *koma* imparts a new perspective, enhances mental reasoning, and fosters considerate behavior among initiates, as evidenced by the below:

“*Being uninitiated, one can see that a person either went through the process of koma or not; koma matures an initiate such that you can distinguish an uninitiated person. Koma imparts the right maturity…and gives a person a new perspective. When initiated, you become considerate*” (86 years old widowed pensioner, P1-FGD)

*Participant 1: The mental reasoning and capacity of an initiated maiden will always prove that a person went through the process of koma.* (83 years old widowed pensioner, KII)

Moreover, participants highlight the cognitive and psychological benefits of the initiation process, noting that it equips initiates with the ability to understand personalities, think critically, and make informed decisions. This suggests that *koma* [] serves as a form of psychological maturation, enabling initiates to navigate the complexities of adult life with wisdom and maturity. The discussion further emphasizes the perceptible differences in thinking patterns and behavior between initiated and uninitiated individuals, with initiated women perceived as mentally balanced and mature as per the below:

“*Initiated people learn personalities and understand them, and they do not do things without thinking, and we are psychologically mature… initiation process matures a person*.” (45 years old, married, Social Worker, KII)

“*It matures you as a person; you will see a difference when you are from initiation process; you see yourself as a grown woman”* (23 years old, matriculated, unemployed, KII)

“*She (referring to the initiated maiden) is mature… the initiated women are balanced mentally*.” (78 years old, widowed, pensioner, KII)

*“Uninitiated maiden and the initiated ones differ concerning thinking patterns; once you go through the initiation process, even your thinking changes…your perceptions change.”* (71 years old, married, pensioner, KII)

“*You can be wiser, but the mind of the initiated and the uninitiated are not the same…you can see by the level of thinking…you can detect that this one is initiated like me. This one is not; you can detect that she is immature…they are not on the same level; the uninitiated speak without thinking.*” (72 years old, widowed, pensioner, KII)

“*Initiation process makes one to gain insight.*” (85 years old, widowed, pensioner, P2-DD)

“*It imparts a balanced thinking*.” (81 years old, widowed, pensioner: P1:DD)

The above finding emphasizes the significance of traditional female initiation as a transformative rite of passage that shapes initiates’ mental and psychological wellbeing. The participants’ accounts provide valuable insights into the perceived effects of *koma* on cognitive development, highlighting its role in fostering maturity, balanced thinking, and insight among initiates. These findings contribute to a deeper understanding of the psychological significance of female traditional initiation within the context of cultural practices and rites of passage.

#### 3.2.2 *Koma* as a catalyst for cultural identity formation

Identity formation is a complex and ongoing process through which individuals develop a sense of who they are and where they fit in the world. It involves exploring and integrating various aspects of one’s identity, including cultural, ethnic, religious, gender, and personal identity. This process is influenced by interactions with family, peers, society, and culture, as well as personal experiences and self-reflection. Identity formation typically begins in adolescence but continues throughout life as individuals encounter new experiences and challenges. It plays a crucial role in shaping beliefs, values, attitudes, and behavior, influencing how individuals perceive themselves and others. A strong sense of identity provides individuals with a foundation for self-confidence, resilience, and meaningful engagement with the world around them. They contribute to a sense of purpose, fulfillment, and social integration, allowing individuals to navigate life’s challenges and form meaningful relationships.

The excerpts from the participants reveal the profound impact of traditional female initiation on young women’s social belonging and peer relationships. Participants’ account illustrates the feelings of exclusion and envy experienced by those who have not undergone the initiation process. She expresses a strong desire to participate in initiation rites to align herself with her peers and bridge the perceived gap between herself and the initiated women in her community. This sentiment is echoed by one participant, who emphasizes the importance of initiation in fostering a sense of belonging and inclusion within the community. The desire to conform to societal norms and expectations is further highlighted by a participant who views initiation as an obligation to maintain social cohesion and harmony with her peers.

Additionally, the participant’s statement emphasizes the ambitious aspect of female traditional initiation, with initiates serving as role models for younger women in the community. The initiation process is seen as a rite of passage and a means of acquiring status and identity within the social hierarchy. Furthermore, the observation made by the Participant highlights the social segregation that occurs between initiated and uninitiated individuals, with the former forming exclusive social circles and distancing themselves from those who have not undergone the initiation process.

“*Ahaa… I used to be envious of seeing people who went through the initiation process, especially my age group, coming back with their initiation names. I still used my childhood name and could not even play with them. Can you imagine? Because there is a distance, like when you are with them, you are not in their category. That is why I desired to go through the initiation process. So that I could be like other women. What was it exactly that they were hiding from me? What was happening? Because my age group went through the initiation process at (xxx- location)*. *So, I could not go there, but we were no longer playing together when they returned. We were playing separately as they were isolating themselves from us. I could not reach out to them, so I went through the initiation process so that I am able to play with them.*” (23 years old, matriculated, unemployed, KII)

Some participants expressed themselves as follows:

*“It must always be there; it would not be possible that others go through it and others do not… so that they belong”*… (71 years married pensioner, KII)

“*I also wanted to be like that woman and make the beads she wore on her neck and hands with traditional bracelets on her feet.*” (72 widowed pensioner, KII)

“*Just because my peers went through the initiation process and my elder sisters also went, I too had to follow suit, and it was like an obligation because if I did not go, I would have forfeited the sense of belonging with my peers.*” (76 years old widowed, pensioner, KII)

“*You do not mix with them anymore …you associate only with the initiated now*” (76 years old, widowed, pensioner, KII)

This reveals the multifaceted interplay between traditional initiation, social identity, and peer relationships among young women. Female traditional initiation is a cultural practice and a mechanism for shaping social dynamics and reinforcing community cohesion. The desire for inclusion and belonging drives many young women to participate in the initiation rites, highlighting the deep-rooted significance of this tradition in shaping social norms and interpersonal relationships within the community.

#### 3.2.3 *Koma* and perseverance

Perseverance is a fundamental trait that empowers individuals to overcome obstacles, navigate challenges, and achieve their goals despite setbacks or adversity. It encompasses determination, resilience, and steadfastness in pursuing one’s aspirations despite difficulties or failures. It instills a resilience mindset, enabling individuals to bounce back from setbacks, learn from their experiences, and adapt to changing circumstances.

The statements provided by the participants highlight the theme of perseverance instilled through the female traditional initiation process, emphasizing how *koma* instills perseverance in individuals. This sentiment is echoed by the participant, who credits *koma* for enabling her to endure challenges within her marriage, illustrating how perseverance is essential for navigating difficult circumstances. The belief is that going through the process of initiation rites teaches individuals to endure pain and develop perseverance. This sentiment is further reinforced by reflecting on the challenging rituals of initiation and the importance of persevering despite fear and discomfort. The participants also highlight the transformative nature of *koma*, where individuals undergo physical and emotional challenges to emerge stronger and more resilient. The participant acknowledges the role of perseverance in overcoming obstacles and preparing for the responsibilities of marriage.

The following expressions about perseverance were made:

*“It inculcates perseverance in a person.”* (83 years old widowed pensioner, KII)

“*It helped me with staying with my in-laws that even when you are being ill-treated, you persevere because you are an initiated and married woman; you cannot just pack your belongings and go back to your mother’s house because you are suffering… Its imparted perseverance, and I stayed in my marriage despite challenges. As they say, a woman’s grave is with her in-laws*.” (75 years old widowed pensioner, KII)

*“I can persevere; I do not know if anyone can persevere while she is not initiated. I understand that even if there is no food in the house, I must improvise if there is a maize meal I can cook so that people in the home can eat… Female traditional initiation is just another platform for teaching women how to endure pain… it teaches you perseverance.”* (33 years old, unmarried, matriculated, unemployed, KII)

*“I think koma teaches us not to give up and always take chances because there were brutal rituals like stepping on blazing wood…we were scared, but you had to do it and persevere all the time. I think we have learned perseverance… I have learned that one must persevere despite how difficult life can be.”* (24 years old, unmarried, unemployed graduate, KII)

*“You persevere at the end of the day; you will realize when you go through thojana [A graduation-equivalent ceremony held upon completion of the initiation process], we go there during the early hours of the morning naked…you persevere the cold, but at the end of it, when the sun rises, they sing hwibilahwibila[graduation song], it is now pleasant you wear thlapetsana[pigeon’s feather- symbolizing a new beginning]and beads, and you look good… you persevere through the difficulties of all these months… they prepare you for marriage so you can persevere and always think on your feet.”* (23 years old, married Personal Assistant, KII)

Lastly, the perspective provided also reinforces the significance of perseverance in maintaining relationships. The 70-year-old woman suggests that individuals may be more inclined to give up on marriages without perseverance, indicating perseverance’s vital role in sustaining relationships and overcoming adversity. These insights illustrate how koma serves as a platform for instilling perseverance, resilience, and determination in individuals, which are essential qualities for navigating life’s challenges as evidenced by the extract below:

*“If there is no perseverance, you can decide to divorce. You do not even think twice or care if you have children together.”* (70 years old widowed pensioner, P4, FGD)

#### 3.2.4 *Koma* and tolerance

Participants described how the initiation process (*koma*) instilled in them a deepened capacity for tolerance and resilience in the face of interpersonal challenges, particularly within marriage. Rather than reacting impulsively or emotionally, some participants demonstrated a reflective stance toward difficult situations, linking their current coping strategies to their initiation training.

One participant, a 23-year-old married personal assistant, noted:

*“Now, in marriage, you will find the in-laws making your life difficult, but because you have learned to handle difficulties during koma, the treatment does not matter much as you console yourself that this too will pass the same way koma did.”* (23 years old married Personal Assistant)

This suggests a learned ability to endure and reframe challenges without being overwhelmed by them. Similarly, an 81-year-old widowed pensioner in a focus group discussion shared:

*“There is no tolerance, but if I can go through the initiation process, then I will have tolerance towards my husband*.” (81 years old widowed pensioner-P3: FGD)

These accounts exemplify how the initiation process is perceived not only as a rite of passage but also as a psychological and emotional foundation that enables women to exercise self-control, patience, and empathy, all of which are central to the broader concept of tolerance.

## 4 Discussion

The paper aimed to explore the psychological impact of traditional female initiation on women who had undergone the process. It emerged from participants that traditional female initiation ceremonies play a significant role in maturing the initiates. These initiation rituals mark the transition from girlhood to womanhood and are often accompanied by various rites of passage designed to instill maturity and responsibility in the initiates. This finding confirms those conducted amongst other tribes which revealed shown that participating in traditional initiation ceremonies contributed to the maturity of the initiates (Matobo et al., 2009). By undergoing these rites of passage, young women develop a deeper understanding of their adult roles and responsibilities, contributing to their overall maturity and development. Talakinu (2023) further suggest that the process of developing a maturity amongst initiates begins during the initiation process where females are presented with the challenges and tasks aimed at encouraging personal growth, resilience, and adaptability, all essential components of maturity (Ofori, 2023; Weichold et al., 2023).

The current study also found *koma* to be significant in instilling perseverance among the initiates. Through various challenges, tests, and teachings during the initiation process, young women learn to endure and overcome obstacles, developing a sense of resilience and determination (Ezenweke, 2016; Setlhabi, 2014; Abbey and Nasidi, 2023). This perseverance extends beyond the initiation period and becomes a valuable trait that empowers women to navigate the challenges of adulthood and societal expectations. The experiences gained during the initiation rites contribute to the development of mental fortitude and the ability to persist in the face of adversity, shaping the character of the initiates and preparing them for the responsibilities and roles associated with womanhood. On the contrary, as argued by Schroeder et al. (2022) coming of age traditions can be challenging to some initiates as some are groomed to assume new adult responsibilities, and sometimes such experiences and even the initiation process negatively affects their physical, emotional, and psychological health and wellbeing.

The study also revealed that *koma* is crucial in instilling tolerance among initiates. Through various rituals, teachings, and experiences during the initiation process, young women are exposed to different perspectives, beliefs, and cultural practices, fostering an understanding and acceptance of diversity within their communities (Munthali and Zulu, 2007; Muriithi, 2023). Initiates learn to respect and tolerate individuals with differing backgrounds, beliefs, and traditions, promoting harmony and cooperation within their social networks. This cultivation of tolerance benefits the initiates personally and promotes peace and unity within their communities, fostering mutual respect and understanding among community members. However, some rites of passage can have detrimental effects and disturb one’s peace as they may be harmful to the mental health of initiates (Schroeder et al., 2022).

The current study found that *koma* serves as transformative events that contribute significantly to forming the cultural identity of initiates. These rites mark the transition from adolescence to adulthood and play a pivotal role in shaping young women’s cultural, social, and personal identities. Research indicates that participation in female initiation ceremonies gives initiates a sense of belonging and connection to their cultural heritage, fostering a strong sense of identity (Mbiti, 1975) (Mann-McFarlane, 2020, unpublished Doctoral Thesis).^1^ Through these rites, initiates are immersed in their community’s traditions, values, and beliefs, which helps them develop a coherent sense of self within their cultural context (Mntambo, 2020, unpublished master’s dissertation)^2^ (Adal et al., 2021). Additionally, initiation rites often involve symbolic rituals and teachings that emphasize the roles and responsibilities expected of women in society, further contributing to their identity formation (Ezenweke, 2016; Talakinu, 2023) (Mann-McFarlane, 2020, unpublished Doctoral Thesis, see footnote 1). While participants in this study largely emphasized the positive role of female initiation in reinforcing cultural identity and imparting valued norms, the discussion must also consider existing literature that highlights potential negative implications. For example, Tamale (2008) argues that some females may undergo initiation due to social pressures or lived experiences that limit their agency, and that the connection between cultural practice and identity is not always straightforward. Although participants did not explicitly describe harmful or distressing elements in their accounts, this absence does not negate the documented risks and critiques in other contexts. The findings thus both align with the literature in recognizing initiation as a culturally significant process and highlight the need for ongoing reflection and safeguards to ensure that agency, consent, and wellbeing are upheld, particularly given the potential for coercion or stigma.

Considering the complexities explored, this study emphasizes the importance of moving beyond critique to offer constructive, culturally sensitive pathways that safeguard young women’s rights while respecting tradition. Integrating traditional practices into policy in ways that uphold health, equity, and dignity requires inclusive community dialogue, robust research, and youth-centered programs that build agency and resilience. By highlighting the value of community-led oversight and culturally grounded education, the study demonstrates how traditional rites can evolve to support the wellbeing of young women. These findings inform practical, community-driven approaches that balance cultural continuity with the promotion of human rights and offering a framework for sustainable, respectful development rooted in both tradition and empowerment.

## 5 Limitations

This study’s findings are shaped by several limitations. This research delved into the psychological significance of *koma ya basadi* as perceived by initiated women. The study exclusively examined women who had undergone the initiation process. Women who had not experienced initiation were excluded from the study, and their perspectives were not considered. The study encompassed women between 16 and 90, with those below 16 and above 90 not included, leaving their viewpoints undocumented. Additionally, both initiated and non-initiated men were ineligible for participation, as the study was centered on women. The participant pool was largely made up of elderly women, offering valuable insight into traditional understandings of *koma ya basadi*, while younger voices were underrepresented. Although their perspectives aligned with those of older participants, this imbalance may limit the study’s ability to reflect evolving interpretations. The small, self-selecting sample also raises concerns about representativeness and potential response bias. In some cases, the recency of initiation may have influenced how openly participants shared their experiences. Despite these limitations, the study provides rich, contextually grounded insights into the psychological and cultural significance of initiation. Broader future research could enhance both depth and reliability.

## 6 Conclusion

This study affirms the complex and deeply rooted cultural significance of traditional female initiation practices, particularly *koma ya basadi*, as rites of passage that promote identity, social belonging, and intergenerational continuity. Consistent with existing literature, findings highlight how these ceremonies transfer cultural knowledge, foster resilience, and prepare women for adulthood through structured rituals that are seen as character-building. These practices remain especially meaningful in rural or historically marginalized communities where tradition serves as a strong social anchor.

By capturing the voices of mainly older women, the study provides valuable historical and cultural framing, yet it also acknowledges the underrepresentation of younger perspectives. This gap points to the need for intergenerational dialogue and further exploration of how meanings and experiences of initiation may be shifting.

Ultimately, the study contributes to a more nuanced understanding of traditional initiation, encouraging a culturally respectful yet critical approach that balances heritage with evolving ideas around health, rights, and agency in contemporary South Africa.

## Data Availability

The raw data supporting the conclusions of this article will be made available by the authors without undue reservation.

## Footnotes

^1^Mann-McFarlane, S. (2020). Rites of Passage Influence on Cultural Identity and Learning Among Emerging Black/African American Female Adults, unpublished Doctoral Thesis, Penn state University Libraries.

^2^Mntambo, L. N. (2020). Umemulo and Zulu girlhood: From preservation to variations of ukuhlonipha nokufihla (respect and secrecy) (Unpublished master’s dissertation). Rhodes University, Makhanda, South Africa.

## References

[ref1] Abbey E. A. Nasidi N. A. (2023). Krobo girls and Dipo puberty rites of passage in the eastern region of Ghana. Int. J. Mod. Anthropol. 2, 1110–1127. doi: 10.4314/ijma.v2i19.4

[ref2] Adal D. B. Robso M. Digile H. C. (2021). Indigenous education system: - some notes on the Guji Oromo age old traditional education practices, southern Ethiopia. Palarch’s J. Archaeol. Egypt Egyptol. 18, 4093–4110.

[ref3] Adeoye-Olatunde O. A. Olenik N. L. (2021). Research and scholarly methods: semi-structured interviews. J. Am. Coll. Clin. Pharm. 4, 1358–1367. doi: 10.1002/jac5.1441

[ref4] Barth F. (1987). Cosmologies in the making: a generative approach to cultural variation in Inner New Guinea. Issue 64 of Cambridge studies in social and cultural anthropology. Cambridge: Cambridge University Press.

[ref5] Creswell J. W. Creswell J. D. (2017). Research design: qualitative, quantitative, and mixed methods approaches. Thousands Oaks, California: Sage Publications.

[ref6] Ezenweke E. O. (2016). The role of initiation rites in socialization and identity formation among the Igbo of Nigeria. Afr. J. Psychol. Study Soc. Issues 19, 77–87.

[ref8] Frechette J. Bitzas V. Aubry M. Kilpatrick K. Lavoie-Tremblay M. (2020). Capturing lived experience: methodological considerations for interpretive phenomenological inquiry. Int J Qual Methods 19, 1–12. doi: 10.1177/1609406920907254

[ref9] Guest G. Bunce A. Johnson L. (2006). How many interviews are enough? An experiment with data saturation and variability: an experiment with data saturation and variability. Field Methods 18, 59–82. doi: 10.1177/1525822X05279903

[ref6001] Guest G. Namey E. E. Mitchell M. L. (2013). Collecting qualitative data: A field manual for applied research. London: Sage Publications.

[ref10] Johnson J. (2011). Through the liminal: a comparative analysis of communitas and rites of passage in sport hazing and initiations. Can. J. Sociol. 36. doi: 10.29173/cjs8650

[ref11] Kangwa K. (2011). Reclaiming the values of indigenous female initiation rites as a strategy for HIV prevention: a gendered analysis of chisungu initiation rites among the Bemba people of Zambia. (Master’s thesis): University of KwaZulu–Natal.

[ref12] Lebese R. T. Mothiba T. M. Mulaudzi M. T. Mashau N. S. Makhado L. (2022). “Rite of passage: an African indigenous knowledge perspective” in Working with indigenous knowledge: strategies for health professionals. eds. Mulaudzi F. M. Lebese R. T. (Cape Town: AOSIS Books), 51–68. 38446972

[ref9006] Matobo T. A. Makatsa M. Obioha E. E. (2009). Continuity in the traditional initiation practice of boys and girls in contemporary Southern Africa Society. Stud. Tribes Tribals 7, 105–113.

[ref13] Mbiti J. S. (1975). African religions and philosophy. London, United Kingdom: Heinemann Educational Books.

[ref16] Morgan D. L. (1996). Focus Groups. Ann. Rev. Sociol. 22, 129–152. doi: 10.1146/annurev.soc.22.1.129

[ref17] Munthali A. C. Zulu E. M. (2007). The dynamics of gender and social cohesion in rural Malawi. J. South. Afr. Stud. 33, 845–860. doi: 10.1080/03057070701691760

[ref18] Muriithi E. N. (2023). A comparative analysis of Aembu and Anglican rites of passage for child socialization in Kigali archdeaconry Embu county, (Unpublished doctoral): Kenyatta University.

[ref9002] Nanegbe E. K. N. (2016). Transitions and transactions: Adult identity development among adolescent girls of the Krobo ethnic group (Master’s thesis, The University of Bergen).

[ref9003] Nyeseh Ofori K. Mohangi K. (2024). Adolescent psychosocial identity development associated with traditional rituals and rites of passage in the Bosomtwe District of Ghana. Vulnerable Child Youth Stud. 19, 733–750.

[ref9004] Ofori K. N. (2023). Adolescent psychosocial identity development associated with traditional rituals and rites of passage in the Bosomtwe District of Ghana (Master’s thesis). University of South Africa. Available online at: https://ir.unisa.ac.za/handle/10500/31295

[ref19] Schroeder E. Tallarico R. Bakaroudis M. (2022). The impact of adolescent initiation rites in east and southern Africa: implications for policies and practices. Int. J. Adolesc. Youth 27, 181–192. doi: 10.1080/02673843.2022.2052123

[ref20] Setlhabi K. G. (2014). The politics of culture and the transient culture of bojale: bakgatla-baga-kgafela women’s initiation in Botswana. J. South. Afr. Stud. 40, 459–477. doi: 10.1080/03057070.2014.913424

[ref21] Sewu N. D. (2023). Relevance of the performance of the dipo rite among the people of Yilo and lower Manya Krobo in the eastern region of Ghana. South Asian J. Soc. Sci. Human. 4, 98–124. doi: 10.48165/sajssh.2023.4308

[ref22] Shoko T. (2009). Komba: Girls initiation rites and enculturation among the VaRemba of Zimbabwe. Studia Historiae Ecclesiasticae 35, 31–45.

[ref6002] Talakinu C. M. (2023). Rethinking female rites of passage: the chinamwali, male power, and african feminisms. J. Hum. 31, 1–21.

[ref23] Tamale S. (2008). The right to culture and the culture of rights: a critical perspective on women's sexual rights in Africa. Feminist Leg. Stud. 16, 47–69. doi: 10.1007/s10691-007-9078-6

[ref9005] Thompson Burdine J. Thorne S. Sandhu G. (2021). Interpretive description: A flexible qualitative methodology for medical education research. Med. Educ. 55, 336–343. 32967042 10.1111/medu.14380

[ref26] Turner V. T. (1967). The forest of symbols: aspects of Ndembu ritual. Ithaca: Cornell University Press.

[ref27] Van Gennep A. (1960). The rites of passage. Chicago: University of Chicago Press (Original French Version (1909) Les Rites de Passage. Nourry, Paris.

[ref29] Weichold K. Mahama S. Fehmer N. (2023). Initiation ceremonies and rites of passage. Available online at: https://www.profpsy.uni-jena.de/profpsymedia/dokumente/weichold-mahama-fehmer-in-press-initiation-ceremonies-and-rites.pdf (Accessed April 3, 2024).

